# Virginia Dental Society

**Published:** 1867-05

**Authors:** 


*Virginia Dental Society.*—A short time since we received from the Secretary, Dr. W. Leigh Burton, a copy of the Constitution and By-Laws of this Society, whose object is "to cultivate the Science and Art of Dentistry, and all its collateral branches : to elevate and sustain the profession and character of Dentists : and to promote amongst them improvement, social intercourse and good will." Article IX. of the By-Laws prohibits any member of the Society from taking an office student for a less term than two years; "and he be advised by his preceptor to attend and graduate at a Dental College." This is a praiseworthy effort, and our friends in Virginia have our best wishes for the success of their efforts to elevate the professional standard.

